# Prosthetic Aortic Valve Endocarditis Creeping Into the Paravalvular Space

**DOI:** 10.7759/cureus.35315

**Published:** 2023-02-22

**Authors:** Mohammad Abdel Jawad, Abdullah Abu Kar, Zaher Fanari, Ali Elkharbotly

**Affiliations:** 1 Internal Medicine, Ascension Via Christi St. Francis Hospital, Wichita, USA; 2 Hospital Medicine, University of California San Francisco, San Francisco, USA; 3 Cardiology, University of California San Francisco Fresno, Fresno, USA; 4 Cardiology, Ascension Via Christi St. Francis Hospital, Wichita, USA

**Keywords:** infective endocarditis, transesophageal echo, multimodality cardiac imaging, paravalvular abscess, prosthetic heart valve

## Abstract

Prosthetic valve endocarditis is a devastating infection with a challenging diagnosis and management. Despite advances in its diagnostic modalities, medical, and surgical interventions, prosthetic valve endocarditis still carries high morbidity and mortality rates. Here, we report a case of prosthetic aortic valve endocarditis that progressed to involve the paravalvular space and the importance of multimodality cardiac imaging in the early detection of paravalvular complications.

## Introduction

Prosthetic Valve Endocarditis (PVE) accounts for twenty percent of all cases of infective endocarditis (IE) [1]. The most common causative microorganism is Staphylococcus aureus, responsible for one in four cases of PVE [1]. The prosthetic aortic valve is responsible for ~ 70% of PVE cases [1]. Early PVE occurs within the first 12 months of prosthetic valve placement. Infection that occurs after 12 months of placement is defined as late PVE. The modified Duke criteria can be used to establish PVE diagnosis [2]. Transesophageal echocardiography (TEE) is essential in diagnosing and managing PVE [3]. Early detection of PVE and the initiation of treatment are essential to avoid complications that carry a worse prognosis. Management of PVE is complex and requires a multidisciplinary team approach. Here, we report a case of prosthetic aortic valve endocarditis that was complicated by paravalvular abscess and discuss how utilization of multimodality cardiac imaging could have resulted in earlier detection of this complication.

## Case presentation

**Figure 1:**
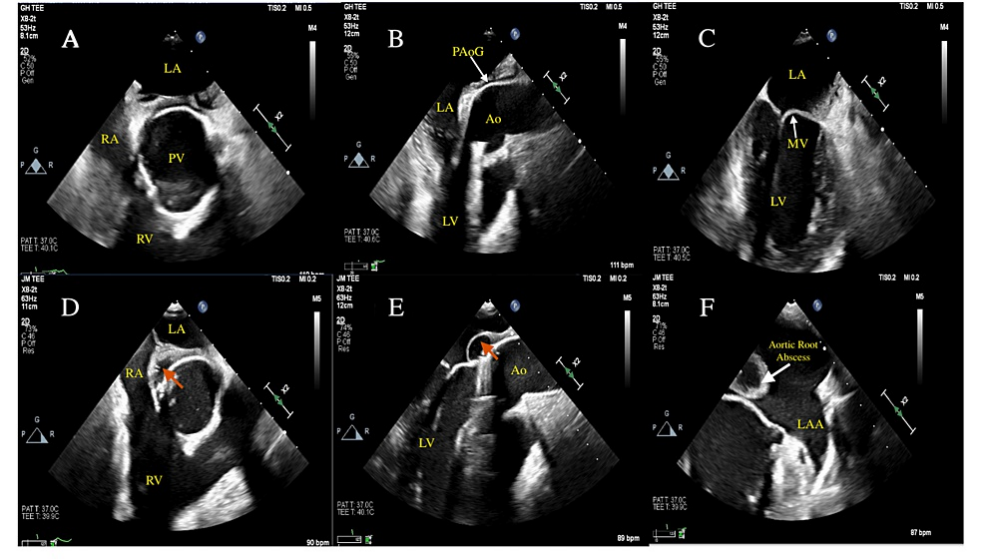
Invasion of the infection into the paravalvular structures (A-C) Transesophageal echocardiography images at initial diagnosis. (A) Short axis view across the prosthetic aortic valve (PV), right atrium (RA), right ventricle (RV). (B) Aorta (Ao) and prosthetic aortic graft (PAoG) can be seen in this view. (C) Mitral valve (MV), left atrium (LA), and left ventricle (LV) can be seen in this two-chamber view. (D-F) Transesophageal echocardiography after five weeks of intravenous antibiotics. Paravalvular abscess (red arrow) can be seen in (D) and (E). (F) Aortic root abscess can be seen in very close proximity to the MV, left atrial appendage (LAA) with no evidence of filling defects.

A 36-year-old male with a history of bicuspid aortic valve status post mechanical aortic valve replacement five years prior to this hospitalization presented to the hospital with altered mental status and fever. The patient denies illicit drug use. Vital signs on admission: temperature of 38-39.5 C; heart rate of 120-150; respiratory rate of 20-35; blood pressure of 120s/80s; and oxygen saturation of 92%-94% on room air. An electrocardiogram showed sinus tachycardia. Lab testing demonstrated elevated white blood cell count and troponin level. Brain magnetic resonance imaging showed acute ischemic bilateral infarcts concerning for embolic source. Blood cultures on admission grew methicillin-sensitive Staphylococcus aureus. The patient underwent TEE that showed vegetation on the aortic side of the mechanical aortic valve (Figures 1A-1C). Prior to surgical intervention, the patient developed a hemorrhagic conversion of his ischemic stroke that required an emergent craniotomy which subsequently delayed his surgery for six weeks. In the interim, the patient was started on intravenous Nafcillin. Repeat TEE after five weeks of intravenous antibiotics showed a new aortic root paravalvular abscess not present on the initial TEE (Figures 1D-1F). The patient subsequently underwent surgical aortic valve replacement with the evacuation of the aortic root abscess. The patient tolerated the procedure well and was discharged to a rehabilitation facility. The patient was seen in the clinic six weeks after discharge from the rehabilitation facility and he was doing well with no residual complaints.

## Discussion

PVE is a serious condition with a challenging diagnosis and management. It has an incidence of 0.3%-1.2% per patient-year [1]. PVE has a heterogeneous presentation, such as fever, heart failure, stroke, or systemic embolization other than stroke. Abscess is the most common paravalvular complication in patients with IE with a prevalence of 14%-22% [4,5]. Compared to patients with native aortic valve endocarditis, patients with PVE have a higher incidence of periannular abscess (40% versus 19%, p <0.001) [5]. Current guidelines recommend a multidisciplinary team approach in patients with PVE [3]

To initiate appropriate therapy, early diagnosis of PVE is critical. Prognosis depends on a timely diagnosis and a tailored therapy that includes antibiotic treatment and when indicated, early cardiac surgery. The diagnosis of PVE is based on clinical manifestations, blood cultures to identify the causative organism, and cardiac imaging. Echocardiography plays an important role in the diagnosis of PVE. It constitutes one of the two major diagnostic criteria in the modified Duke criteria [2]. TEE is the preferred initial diagnostic modality in patients with suspected PVE. Transthoracic echocardiography images are limited by the prosthetic valve components [6]. TEE findings that support PVE diagnosis include oscillating intracardiac mass or vegetation, new periprosthetic leak, prosthetic valve partial dehiscence, new valvular regurgitation, and an annular abscess. In addition to these findings, the detection of periannular complications including paravalvular abscess is essential. The presence of paravalvular abscess is associated with worse outcomes and increased mortality [7].

TEE has high sensitivity and specificity to detect vegetation [8]. However, the sensitivity of TEE for the detection of paravalvular abscesses associated with endocarditis is 87% [9]. This is partly due to artifacts caused by the acoustic shadowing of the prosthetic material. Recent advances in cardiac imaging modalities have resulted in better visualization of the perivalvular space. Specifically, multidetector computed tomography (MDCT) has better spatial resolution and is not affected by the acoustic shadowing of the prosthetic material. This results in better characterization and detection of perivalvular lesions including paravalvular abscesses [8]. Other alternate diagnostic modalities that might facilitate earlier detection of perivalvular complications include fluorine-18-fluorodeoxyglucose (FDG) positron emission tomography (PET)/computed tomography (CT) and cardiac MRI [10].

This case shows the progression of the infection affecting the prosthetic aortic heart valve into the surrounding structures while receiving the appropriate antibiotics. This case highlights the importance of repeat cardiac imaging (TEE) and the implementation of multimodality cardiac imaging in patients with PVE to detect complications that carry a worse prognosis. The first TEE did not show evidence of paravalvular abscess; however, we cannot entirely rule out its presence as TEE is 86% sensitive for detecting paravalvular abscess [8]. This sensitivity increases to 100% if cardiac multidetector computed tomography is done in addition to TEE [8]. Early detection of these life-threatening periannular complications has important clinical implications for patient management and outcomes.

## Conclusions

Echocardiography is an essential diagnostic tool in diagnosing PVE. However, it can miss life-threatening paravalvular complications. Utilization of multimodality cardiac imaging in patients with PVE improves the sensitivity for detecting paravalvular complications that carry a worse prognosis.
